# Third Annual Meeting of the Virginia Society of Surgeon Dentists

**Published:** 1845-12

**Authors:** 


### Miscellaneous Notices.

*Third Annual Meeting of the Virginia Society of Surgeon Dentists.*—We have received, in pamphlet form, a printed copy of the proceedings of the third annual meeting of the Virginia Society of Dental Surgeons, which, agreeably to request, we copy into the Journal. From these proceedings it will be seen that this society was instituted December 12th, 1842, and chartered by the legislature of Virginia, February 3d, 1845.

As we publish the proceedings entire, we do not think it necessary to say much, by way of comment, concerning them. There is one resolution, however, connected with them, which we ought not to pass by in silence, as it was evidently intended as a reflection upon that part of the proceedings of the last meeting of the American Society of Dental Surgeons, concerning the requirements of its members in relation to the use of mineral paste, in filling teeth. Now, how the American Society will be able to survive the shock of so tremendous a rebuke, we confess, we are unable to imagine, nor shall we pretend to say how far the Virginia Society transcended the limits of its own legitimate sphere of action in thus indirectly preferring so grave a charge against it. We are of the opinion, however, that the resolution was wholly uncalled for, and gratuitous on the part of our Virginia brethren. If they disapproved of the action of the American Society, they should have been satisfied with the passage of the resolution declaring the use of amalgam, for filling carious teeth, malpractice. In requiring of its members a mutual pledge to abstain from the use of this article, we contend that the American Society, under the circumstances of the case, exacted nothing more than it had a right to do. Nor should we suppose that any one, for a single moment, would object to give such pledge.

Scattered, as the members of this Association are, over a large portion of the globe, some residing in South America, and others in Europe, there was no other way of reaching them, and ascertaining their opinions and practice in relation to the use of this article. The evil had become a crying one; it was rapidly spreading, and the Society, from every quarter, was loudly called upon to take some decisive action upon the subject. At three successive meetings had it passed resolutions declaring its use for dental purposes, malpractice, but notwithstanding this, many of its members continued to use it. It was therefore imperative on the part of the Society to adopt some strong measures to repel the imputation which was thus crippling its energies and weakening its power to do good. The arraignment and expulsion of one, nor a dozen of its members, would have corrected the evil, so long as others, but against whom positive proof of the fact was wanting, were charged with using the article. To prevent such charges, and at once to free itself from all who persisted in this empirical practice, it adopted the resolutions which have proven so obnoxious to our brethren of the Virginia Society. Had they been present on the occasion, and felt the force of the circumstance, and they would have felt them had they been there, under which the Society acted, we doubt not that every one of them, if they had had the right to have done so, would have voted for the resolutions in question.

Therefore, after having declared the article unfit for dental pnrposes, and the use of it malpractice, if they did not think it necessary to proceed as far as the American Society, in relation to the matter, they should have paused and made themselves acquainted with all the facts and circumstances of the case, before having passed so censorious a resolution against an act of a kindred and elder association, an act which cannot be otherwise than productive of great good, both to the dental profession and community at large.

With regard to the power of the Society to expel a member for non-compliance with these resolutions, the point is so fully discussed and clearly established, by our associate, Dr. Westcott, in the following editorial, which we received after having penned the foregoing remarks, that we do not think it necessary to enter into further argument upon the subject.—*Balt. Ed.*

